# A data-driven approach for motion planning of industrial robots controlled by high-level motion commands

**DOI:** 10.3389/frobt.2022.1030668

**Published:** 2023-01-12

**Authors:** Shuxiao Hou, Mohamad Bdiwi, Aquib Rashid, Sebastian Krusche, Steffen Ihlenfeldt

**Affiliations:** Fraunhofer Institute for Machine Tools and Forming Technology (Fraunhofer IWU), Chemnitz, Germany

**Keywords:** robot motion planning, data driven robot learning, neural network, industrial robot, robot simulation

## Abstract

Most motion planners generate trajectories as low-level control inputs, such as joint torque or interpolation of joint angles, which cannot be deployed directly in most industrial robot control systems. Some industrial robot systems provide interfaces to execute planned trajectories by an additional control loop with low-level control inputs. However, there is a geometric and temporal deviation between the executed and the planned motions due to the inaccurate estimation of the inaccessible robot dynamic behavior and controller parameters in the planning phase. This deviation can lead to collisions or dangerous situations, especially in heavy-duty industrial robot applications where high-speed and long-distance motions are widely used. When deploying the planned robot motion, the actual robot motion needs to be iteratively checked and adjusted to avoid collisions caused by the deviation between the planned and the executed motions. This process takes a lot of time and engineering effort. Therefore, the state-of-the-art methods no longer meet the needs of today’s agile manufacturing for robotic systems that should rapidly plan and deploy new robot motions for different tasks. We present a data-driven motion planning approach using a neural network structure to simultaneously learn high-level motion commands and robot dynamics from acquired realistic collision-free trajectories. The trained neural network can generate trajectory in the form of high-level commands, such as Point-to-Point and Linear motion commands, which can be executed directly by the robot control system. The result carried out in various experimental scenarios has shown that the geometric and temporal deviation between the executed and the planned motions by the proposed approach has been significantly reduced, even if without access to the “black box” parameters of the robot. Furthermore, the proposed approach can generate new collision-free trajectories up to 10 times faster than benchmark motion planners.

## 1 Introduction

**FIGURE 1:**
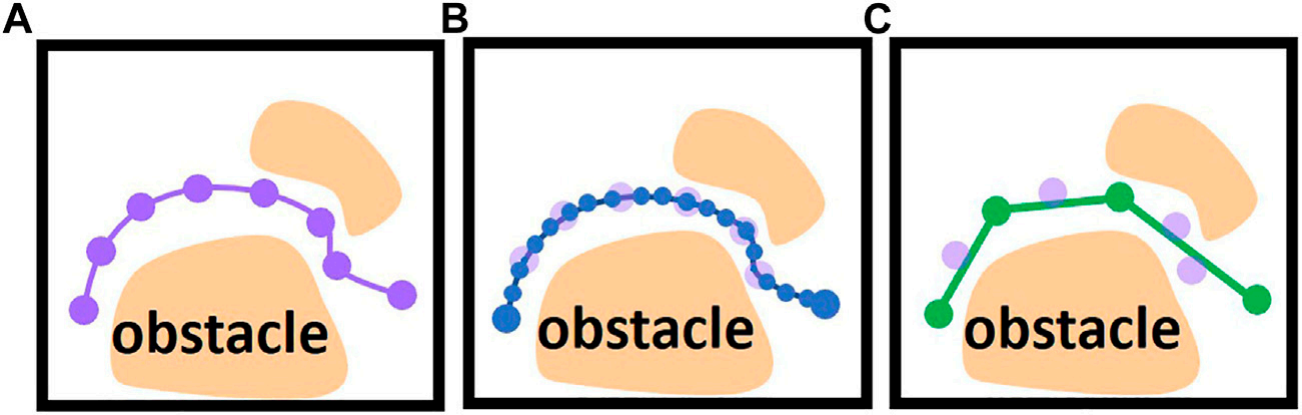
**(A)** Generated trajectory with a large number of *via* points (violet dots). **(B)** An interpolated trajectory interpolated as low-level control inputs (blue dots). **(C)** post-processed trajectory using shortcuts (green dots are reduced *via* points after shortcuts).

Motion Planning is one of the fundamental problems in robotics fields. For decades numerous methods have been proposed for this task by leveraging two common techniques: Optimization-based and heuristic search-based techniques. The trajectories generated by both motion planning paradigms usually include a large number of *via* points (Figure 1A) and require post-processing to deploy to industrial robots.

The trajectories of the industrial robot are typically programmed in the native language of the robot manufacturer. These programming languages pre-define a set of high-level motion commands. The typically high-level motion commands are Point-to-Point, Linear and Cycle motion. The robot control system provided by the robot manufacturer has its own interpolation algorithm and control loop to execute the programmed motion. These control parameters are finely tuned by the robot manufacturer according to the dynamic behavior of each robot and they are usually inaccessible for the user.

There are two ways to plan and deploy robot motion on most control systems of industrial robots.

### 1.1 Planning and deploying robot motion with high-level motion commands

**FIGURE 2:**
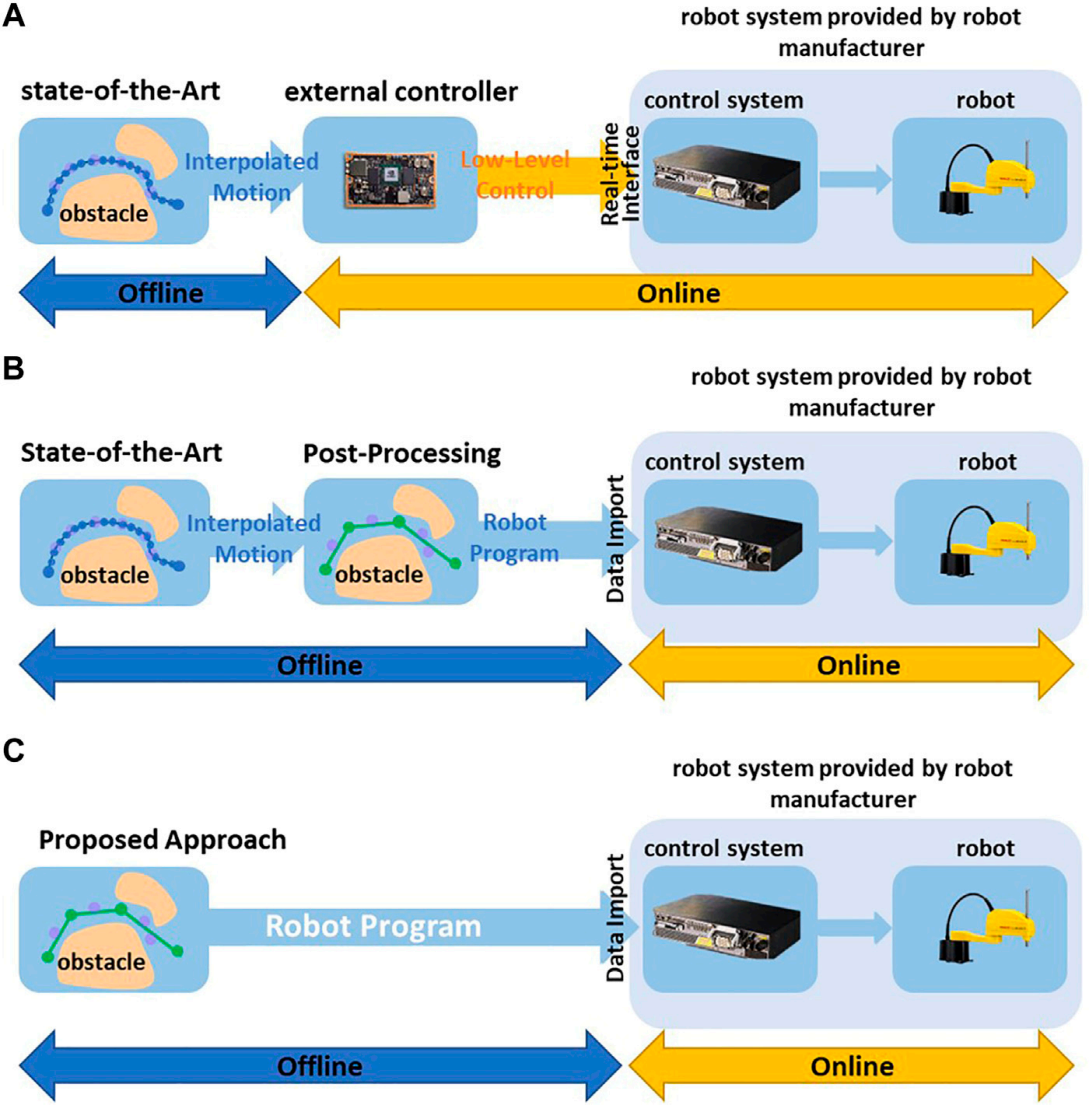
**(A)** Deploying trajectories using post-processing and external control loop. **(B)** Deploying trajectories using post-processing and robot’s own control system. **(C)** Direct Deploying trajectories based on High-Level Motion Commands without post-processing and using robot’s own control system.

For some robot systems, the user can only use the pre-defined high-level motion commands and adapt their parameters to program the desired robot motions, such as programming the start and goal configuration of Point-to-Point motion. In this case, most methods use random shortcuts to reduce the amounts of *via* points. For example (Hauser and Ng-Thow-Hing, 2010), uses various interpolation algorithms, such as parabola and linear interpolation, to directly connect two *via* points on the trajectory. If the direct connection is collision-free, the redundant *via* points can be eliminated (Figure 1C). Since some parameters of the robot are inaccessible, such as dynamic behavior and control parameters, these interpolation algorithms usually use estimated values to interpolate the robot’s motion. Then the post-processed trajectory should be converted to pre-defined high-level motion commands and imported into robot control systems (Figure 2B) in the offline phase. In the online phase, the robot control system provided by the robot manufacturer executes the motion commands. The robot control system uses the interpolation algorithm and control parameters implemented and fine-tuned by the robot manufacturer, which differ from the estimated value used in the offline phase. It may result in a geometric and temporal deviation between the executed and the planned motions. The geometric deviation may cause a collision between the robot and static environments. For example, Figure 1 shows the trajectory planned by the interpolation algorithm used by the shortcut method during offline post-processing, and Figure 2 shows the actual robot motion executed by a real robot control system with the interpolation algorithm implemented by robot manufacturers.

### 1.2 Planning and deploying robot motion with low-level motion commands

Some robot control systems with an additional communication interface allow an additional control loop to command the robots with low-level control inputs in real time, such as position, the velocity of robot joints (yellow arrows in Figure 2A). Most state-of-the-art planners interpolate the motion between the *via* points to low-level control inputs in the offline phase (Figure 1B) and use an additional controller to execute the interpolated trajectories (Figure 2A). (Elhaki and Shojaei, 2022; Rahali et al., 2022; Tan et al., 2023) use various control algorithms in the online phase to minimize the deviation between the executed and the planned motion. However, in heavy-duty industrial robot applications that widely use high-speed and long-distance motions, the deviation becomes significant. For example, in the motion planning framework MoveIt (Chitta et al., 2012), the user should define the maximum jerk and acceleration of joints to interpolate the planned motion in the offline phase. In the online phase, the additional controller tracks the planned motion in real time. If the actual maximum acceleration of the joint during the execution can not reach the values defined by the user, the executed robot motion is slower than planned. This temporal deviation may lead to a collision between the robot and dynamic obstacles such as other robots. For example, in some multi-robot system, the planner schedule multiple robots to pass through a shared area at different timesteps. A robot may collide with others when it enters the shared area earlier or later than planned.

### 1.3 Contributions

The robot motions planned in the described two ways above should be verified in deploying phase to check whether the geometric and temporal deviation between planning and executing of robot motion results in a collision. When the deviation leads to a collision, the actual robot motion must be adjusted and verified again. This process usually iterates manually many times, thus increasing the effort of deploying robot motion.

Most state-of-the-art collision-free motion planning methods focus on improving the performance of motion planning algorithms in the offline phase, such as computation time and success rate of collision avoidance. Today’s agile manufacturing systems require not only automatic robot motion planning but also rapid deployment of robot motions. Therefore, more research is still needed to bridge the gap between offline planning and the rapid deployment of robot motions for reliable online execution. Therefore, we proposed a data-driven motion planning approach that considers deploying and deploying the planned motion already in the offline planning phase. The proposed approach overcomes the problems mentioned above:

(1) The proposed approach uses a neural network structure to simultaneously learn high-level commands and robot dynamics from acquired realistic collision-free trajectories. In the offline planning phase, the trained neural network structure can generate collision-free trajectory as high-level motion commands, such as long-distance, high-speed Point-to-Point and Linear motion. These motion commands can be converted as manufacture-specifical robot language and directly imported into any robot control system (Figure 2C). Because the robot control system can execute these motion commands, the proposed approach does not need an additional control loop to control robot motions in real time and constructs a simpler control architecture. Furthermore, the robot manufacturers tuned the control algorithm of their robot control systems by fully accessing the robot parameters. Therefore, the proposed approach achieves a more stable control structure than the methods described in Figure 2A.
(2) The neural network learns realistic robot dynamics and motion interpolation from actual robot motion execution and uses them to accurately calculate the actual robot motions executed by the robot control system. For example, at each search step in the offline planning phase, the proposed approach use learned robot dynamic behavior to interpolate the robot motion and check whether the robot collides with other obstacles. In the proposed approach, the planned robot motion deviates from the realistic robot motion slightly. Therefore, it can be guaranteed that as long as the robot motion planned offline is collision-free, the robot will also not collide with obstacles as executed by the robot control system. This feature addresses the problem described in Section 1.2. The robot motion planned by the proposed approach does not need to be verified iteratively physically during the deployment phase, thus reducing the manual effort and the time-consuming of the engineering process.

The proposed approach is evaluated on two different industrial applications. The results indicate that the proposed approach can generate high-level motion commands directly deployed to real robot systems with a reduced temporal and spatial deviation between executed and planned motions.

## 2 Related works

### 2.1 Motion planning methods

#### 2.1.1 Optimization-based motion planning methods

Optimization-based motion planning originated in the field of optimal control and has been used for decades in robotics. The trajectories are usually discretized into *via* points, equally spaced in time. The control inputs at every *via* point are considered as optimization variables, such as angle, velocity and acceleration of robot joints. The collision and the kinematics limits of robot joints are modeled as constraint items. The length, smoothness and execution time of trajectory are described as cost functions that should be minimized. Ratliff et al. (2009), Schulman et al. (2013), Zucker et al. (2013), and Schulman et al. (2014) use different approaches to optimize the modeled motion planning problems with various constraints and objectives. The trajectories are usually finely discretized into a large number of *via* points to find valid solutions in complex and high-dimensional solution spaces.

#### 2.1.2 Sampling-based heuristic search methods

In the past several decades, the sampling-based heuristic search method has been widely adopted in the field of motion planning in high-dimensional configuration space with great success. Rapidly-exploring Random Trees (RRT) (LaValle, 1998), optimal Rapidly-exploring Random Trees (RRT*) (Karaman and Frazzoli, 2011), Fast Marching Tree (FMT) (Janson et al., 2015) and their extensions (Kuffner and LaValle, 2000; Karaman and Frazzoli, 2011; Bdiwi et al., 2018; Otto et al., 2021) explore the configuration space incrementally by connecting feasible samples to a search tree. As the complexity of environments and the DOFs (degrees of freedom) of robot increase, samples are often infeasible. Therefore, the number of samples needs to be raised to achieve probabilistic completeness.

Multiple informed methods explore regions with a higher probability of generating feasible paths to improve the searching efficiency in the configuration space of robots. Data-driven techniques such as supervised learning, imitation learning and deep reinforcement learning techniques are quickly becoming useful tools to improve the efficiency of informed searching in high-dimensional configuration space.

##### 2.1.2.1 Learning sampling strategy

Cheng et al. (2020) learns to predict the optimal sampling distribution over low-cost, valid samples. Based on the learned optimal sampling distribution, the classical searching algorithms are used in the planning phase to guide the search progress towards the region with more optimal, feasible paths. Similarly (Gaebert and Thomas, 2022), uses a CVAE Network to learn a sampling strategy that draws samples based on the environment perception to improve sampling efficiency. In the planning phase, the learned adaptive sampling strategy is used with an adaptive probability λ and a uniform sampling with 1−λ . The combination of these two strategies guarantees asymptotic optimality. Instead of implicit learning of sampling distribution (Molina et al., 2020; Shah and Srivastava, 2022), learn to predict critical regions that have a high density of feasible motion plans in the given environments.

##### 2.2.2.2 End-to-end learning low-level control policy

In addition to the learning of sampling strategy (Bhardwaj et al., 2017; Huh and Lee, 2018; Jurgenson and Tamar, 2019; Qureshi et al., 2019; Qureshi et al., 2020; Jinwook et al., 2022), learn to directly generate end-to-end low-level control policy to guide the search progress efficiently towards goal regions. These methods learn search strategies from previous planning problems and apply them to new ones. Qureshi et al. (2019) and Qureshi et al. (2020) designs two neural networks. The first one is embedding the points cloud of the environment into a hidden vector. The second network takes the environment embedding, current state, start and goal state as inputs to generate a sample for the next search step. In (Huh and Lee, 2018), a reinforcement learning approach is proposed. The control actions and corresponding state-action values in a given state can be learned in the learning phase. The trajectory expands towards the goal in the planning phase based on the state-action value of possible control action at each search step. Bhardwaj et al. (2017) defines the search process as a Markov decision process and uses dynamic programming to estimate the cost-to-go value of each possible sample. In (Jurgenson and Tamar, 2019), a modified Deep Deterministic Policy Gradient (DDPG) algorithm is proposed to learn control policy through a trial-and-error fashion, which generates data with a more reasonable distribution, including collision-free expert data and data that escapes the obstacle. Jinwook et al. (2022), new trains a Higher Order Function network to represent the cost-to-go function over the configuration space. In the planning phase, the trained network generates a smooth and continuous cost-to-go function directly from workspace information. The gradient of the cost-to-go function yields continuous collision-free trajectories.

The aforementioned learning-based methods generate low-level control inputs, such as position, the velocity of robot joints. These low-level control inputs should be post-processed to be deployed to real robot systems.

### 2.2 Deploying generated trajectories to robot system

The works mentioned above focus on improving and verifying the performance of collision-free motion planning algorithms in simulation environments rather than on how to deploy the planned robot motion in real robot systems. Bhardwaj et al. (2017); Jurgenson and Tamar (2019), Molina et al. (2020), and Shah and Srivastava (2022) only verify their algorithms in simulation environments. Huh and Lee (2018), Qureshi et al. (2019), Cheng et al. (2020), Qureshi et al. (2020), Gaebert and Thomas (2022), and Jinwook et al. (2022) deploy planned trajectories in real robot systems by using additional controllers to control the robot motion in real time, such as Robot Operation System (Quigley et al., 2009). In these methods, the robots usually run at low speeds to ensure that the robot can precisely track the planned collision-free motion.

Rahali et al. (2022) and Tan et al. (2023) use different algorithms to reduce the motion tracking errors of robots. However, these methods require the robot’s dynamics to be identified and modeled. The algorithms in (Elhaki et al., 2022; Elhaki and Shojaei, 2022) are designed to control multibody systems, such as tractors and underwater vehicles, without requiring detailed system models. Different from these systems, industrial robots have own control systems. Any additional control algorithms must run on an additional controller and control the robot’s motors through an interface provided by the robot own control system (Figure 2A). The stability of this control architecture cannot be guaranteed because some parameters of the internal control loop in the robot control system are not accessible. Furthermore, the communication time between the additional controller and the robot control system also affects the stability and performance of the entire control architecture. For example, the control systems of KUKA heavy-duty robots provide an Ethernet-based communication interface (Robot Sensor Interface- RSI) to control the robot motion using an additional control loop. The cycle time of this communication interface is 4 ms . Therefore, it limits the control algorithms to reduce tracking errors in higher control frequency. In some industrial applications, the high-speed and long-distance robot motions in 4 ms may lead to significant tracking errors.

Again, the methods mentioned above use additional control loops to control the robot in real time to track the motion planned and interpolated in the offline phase. In contrast, the proposed approach does not require an additional control loop to track the planned motion since the proposed approach generates collision-free motions as high-level commands, which can be executed directly by robot control systems with a small deviation from the planned motion of less than .5% on average.

## 3 Problem definition

This section describes the notations used in this work and formally defines the problem we consider.

Let $\chi \subseteq R^{d}$ be the configuration space of a robot system with degrees of freedom d∈N, d>2 . Let $U\subseteq R^{d}$ be the control input space of a robotics system. Let the discrete-time dynamics of the robot be defined by $f_{\chi }$ :

$$\begin{array}{c} x_{k+1}=f_{\chi }\left(x_{k},u_{k}\right) \end{array}$$ (1)

where $x_{k}\in \chi$ and $u_{k}\in U$ denote the state and control input of the system at k -th search step.

In contrast to the approaches described in (Bhardwaj et al., 2017; Huh and Lee, 2018; Jurgenson and Tamar, 2019; Qureshi et al., 2019; Cheng et al., 2020; Molina et al., 2020; Qureshi et al., 2020; Gaebert and Thomas, 2022; Jinwook et al., 2022; Shah and Srivastava, 2022), this work considers the high-level motion commands commonly used in robot handling applications as control inputs $u_{k}$ . These commands typically consist of motion types (such as Point-to-Point, Linear and Circle motion) and motion parameters such as motion velocity and the desired state to be reached.

This work considers static obstacles and dynamic obstacles whose motions are known. For example, for the multi-robot system, motions of all robots are usually planned one by one. When planning the motion of a given robot, the motions of the other robots are known. Let $\chi_{feasible,t}\subseteq \chi$ define the feasible state space of the robotics system, in which the robot did not collide with static and dynamic obstacles at timestep t , $x_{init}\in \chi_{feasible,0}$ the initial state, and $x_{goal}\in \chi_{feasible,t}$ the goal state.

In this work, a trajectory π is defined as a series of states and high-level control commands:

$$\begin{array}{c} \pi =\left(x_{0},u_{0},\;x_{t_{0}},x_{1},u_{1},\;x_{t_{1}},\ldots ,x_{k},u_{k},\;x_{t_{k}}\right) \end{array}$$ (2)

where $t_{k}$ is the timestep of k -th *via* point in the trajectory.

### 3.1 Main problem

For complex environments and robot systems with high DOFs (degrees of freedom), the solution space of the motion planning problem is highly dimensional. Even if the solution space is represented implicitly using the sampling-based technique, it cannot be searched efficiently. In this work, the proposed approach focuses on learning the feasible solution space of motion planning problems from previous experience to improve search efficiency. In other words, the proposed approach first learns to perceive the environment surrounding the robot. Then the robot dynamics are learned to preciously simulate realistic robot motion. At last, the proposed approach learns which optimal high-level commands can move the robot toward to goal region with realistic dynamics in the perceived environment model at each search step.

### 3.2 Subproblem 1: Learning local feasible solution space of motion planning problem

Since learning the complete solution space is very difficult and does not scale well to other problems, our approach begins with learning the local feasible solution space $L_{local}$ :

$$\begin{array}{c} L_{local}\left(x_{k},\phi_{k}|x_{goal}\to u_{k}\right) \end{array}$$ (3)

The locally feasible solution space consists of all feasible control policies that only consider the local system state (e.g., the state of the environment $\phi_{k}$ , the current state of the robot $x_{k}$ and the target state $\chi_{goal}$ ) and guide the robot from the current state toward the target area with control command $u_{k}$ at k -th search step.

### 3.3 Subproblem 2: Suitable representation of dynamic environment

Since this work considers environments with static and dynamic obstacles, the geometric and temporal information of the environment should be represented as environment state $\phi_{k}$ at k -th search step and used in subproblem 1.

### 3.4 Subproblem 3: Learning robot dynamics $f_{\chi }$ controlled by high-level motion commands

The execution time and interpolation of robot motion between two states should be calculated to check the collision between the robot and the obstacles during the transition from one state to the next state at each search step. As mentioned before, the robot dynamics controlled by high-level motion commands are seen as a “black box.” Therefore, the proposed approach learns the realistic robot dynamics controlled by high-level motion commands and uses it to calculate the realistic robot motion.

## 4 Methods

**FIGURE 3:**
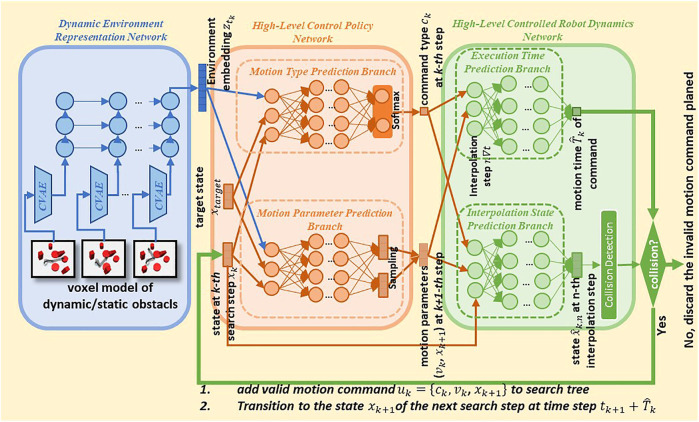
Architecture and planning pipeline of the proposed approach.

The core of the proposed approach is three neural networks (Figure 3) which solve the main problem described in Section 3. Sections 4.1–4.3 describe the functionality of each neural network and how they solve the corresponding subproblems. Then we give an overview of the entire pipeline of the proposed approach in Section 4.4.

### 4.1 Dynamic Environment Representation Network for subproblem 2

Since the motions of the dynamic obstacles are known, the environment can be discretized into a series of frames. The 3D model, such as the voxel model of the environment at each frame, can represent spatial and geometric information.

However, directly using this high-dimensional representation to learn control policy for subproblem 2 leads to a large-scale network that may be difficult to train. Therefore, a separate network struct is used to extract spatial and temporal features of the environment as low-dimensional representation.

Firstly, an encoder embeds the voxel model of the dynamic environment at each discrete timestep into a hidden vector $s_{t}$ . Let denote this embedding as $h\left(\phi_{t}\right)$ , which compresses the spatial state of the dynamic environment $\phi_{t}$ at the timestep t :

$$\begin{array}{c} s_{t}=h\left(\phi_{t}\right) \end{array}$$ (4)

Then an RNN-based encoder embeds the temporally ordered hidden vectors $s_{t},\;s_{t+1}$ …, $s_{t+n}$ into a hidden vector $z_{t}$ , which represents the temporal information of the environment after current timestep t .

$$\begin{array}{c} z_{t}=r\left(s_{t},s_{t+1},\ldots ,s_{t+n}\right) \end{array}$$ (5)

### 4.2 High-level control policy network for subproblem 1

The high-Level Control Policy Network is the core component of the proposed approach. Let denote the high-level control policy network $q_{\theta }$ with its parameter as

$$\begin{array}{c} u_{k}=q_{\theta }\left(x_{k}{,z}_{t_{k}},x_{goal}\right) \end{array}$$ (6)

When the robot arrives a state $x_{k}$ after k -th search step at timestep $t_{k}$ , the network takes the current state $x_{k}$ , the embedding of the dynamic environment $z_{t_{k}}$ at timestep $t_{k}$ and the target region $\chi_{goal}$ as inputs to generate a high-level motion command $u_{k}$ . The high-level motion command $u_{k}$ consists of command type $c_{k}$ and corresponding motion parameters, such as the motion speed $v_{k}$ and the desired state $x_{k+1}$ to be reached.

### 4.3 High-Level Controlled Robot Dynamics Network for subproblem 3

We designed a neural network to predict the dynamics of robots controlled by high-level commands. This network predicates the execution time and interpolation of high-level motion commands.

$$\begin{array}{c} {{\hat{T}}_{k}=f}_{execution\_time}\left(x_{k},u_{k}\right) \end{array}$$ (7)

$$\begin{array}{c} x_{k,n}=f_{interpolation}\left(x_{k},u_{k},t_{k}+n\nabla t\right) \end{array}$$ (8)

where $f_{execution\_time}$ denotes the function to predicate the execution time ${\hat{T}}_{k}$ , $x_{k}$ the current state and $u_{k}$ the motion command, respectively. $f_{interpolation}$ denotes the function to predicate an interpolation state of the robot motion ${\hat{x}}_{k,n}$ at n -th timestep $t_{k}+n\nabla t$ , where ∇t denotes the interpolation resolution.

### 4.4 Robot motion planning with learned feasible solution space

The entire pipeline of the proposed approach consists of the following procedures.

#### 4.4.1 Data collection

Firstly, the expert data for training the above-described neural networks should be collected. The proposed approach requires plenty of realistic data, which are expensive in terms of time and resources. Therefore, the near-realistic simulation environment Visual Components (Visual Components, 2021) are used to generate realistic datasets and verify the planning results. Visual Components contains an offline programming system that can connect with VRC module (Virtual Robot Controller) (Bernhardt et al., 1994). The VRC module integrates the original robot controllers and provides a simulation accuracy of .00005 radians and 1% cycle time. In this work, following data are collected in the simulation environment.

##### 4.4.4.1 Environment data

In offline robot programming, the geometry of the production cell is represented through 3D polygon mesh models in simulation software. The polygon mesh models of the obstacles in the environment are exported and collected as raw data for training Dynamic Environment Representation Network. Then the 3D polygon mesh of obstacles is rasterized into 3D voxel models because 3D voxel grids have a highly regular data format, which is suitable for representation learning. In contrast to representing the environment in point clouds, the resolution of the voxel model can be easily adapted to suit the diverse requirements for environment representation for different robot applications.

**FIGURE 4:**
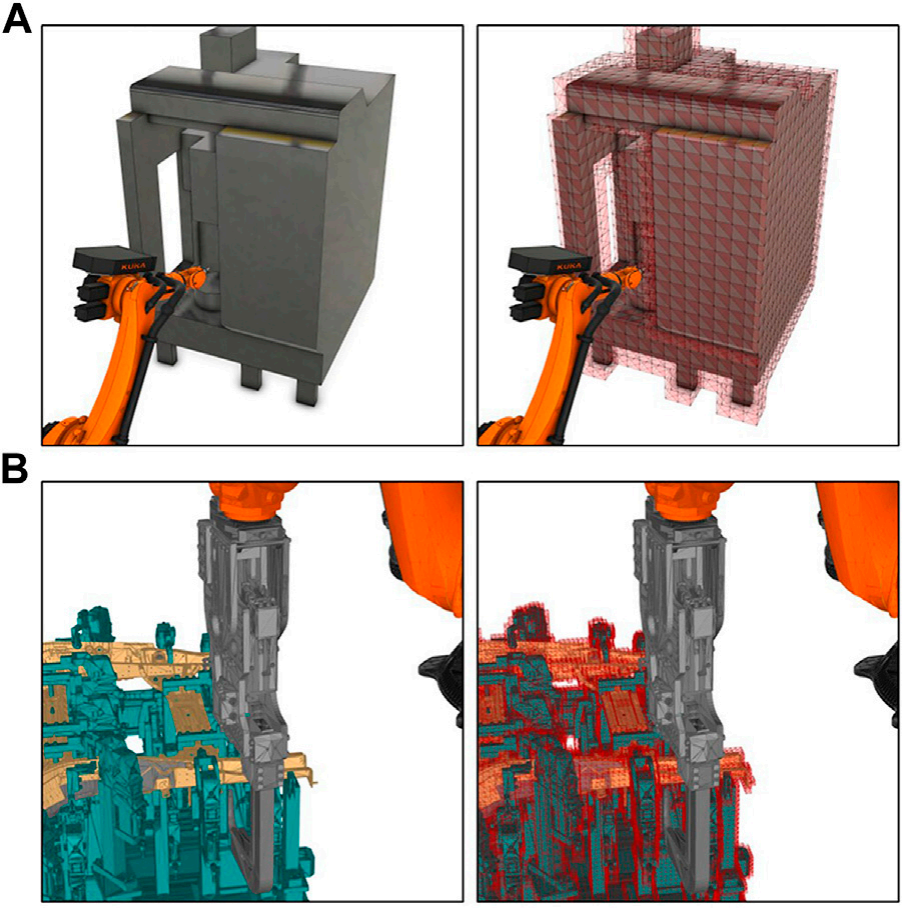
**(A)** Voxel model with low resolution (edge length of each voxel is 8 cm ). **(B)** Voxel model with high resolution (edge length of each voxel is 1 cm ).

For example, in some high-speed handling tasks, the robot should keep a safe distance from the obstacles in the environment. In this case, the edge length of the voxel grids occupied by the obstacles should be increased to leave enough space between the robot and the obstacles (Figure 4A). However, for tasks that require the robot to perform delicate operations, such as spot welding tasks where the welding gun enters some narrow areas, we need to increase the resolution of the voxel models to represent more details of the narrow areas (Figure 4B).

##### 4.4.4.2 Robot programs

Robot programs for scenarios of different applications are collected to learn high-level motion commands. The robot programs consist of high-level motion commands, which are programmed manually or automatically through other motion planners. These robot programs should be executed and verified on real robot systems or near-realistic simulation environments to ensure that the programmed robot motions are collision-free.

##### 4.4.4.3 Realistic robot motions

Training the High-Level Controlled Robot Dynamics Network requires realistic robot motions executed by the robot control system. On the one hand, the collision-free robot motions generated in Section 4.4.1.2 are be reused. On the other hand, more high-level commands are randomly generated. These commands should also be executed on real robot systems or near-realistic simulation environments to collect realistic robot motions.

#### 4.4.2 Model training

In the second procedure, the three neural networks described in Section 4.3 are trained on the data gathered in Section 4.4.1. All three neural networks are trained in an offline supervised fashion. The experiment settings for model training are detailed in Section 5.2.

#### 4.4.3 Offline robot motion planning

In the offline planning procedure, the trained neural network models are used to search a collision-free robot motion from the initial state $x_{init}$ to the goal state $x_{goal}$ . The search process starts from the initial state $x_{k}$ . At each search step k (at the timestep $t_{k}$ ), Dynamic Environment Representation Network embeds the dynamic environment into a low-dimensional hidden vector $z_{t_{k}}$ (see the blue block in Figure 3). $z_{t_{k}}$ is then fed into the High-Level Control Policy Network along with the current state $x_{k}$ and the target state $x_{target}$ to generate a high-level motion command $u_{k}$ consisting of motion command type and motion parameters (see the orange block in Figure 3). The High-Level Controlled Robot Dynamics Network then takes $u_{k}$ as input to predict all interpolation states $x_{k,n}$ and execution time ${\hat{T}}_{k}$ of the motion to the next search step. For each interpolation state, the collision between the robot and obstacles is checked using conventional forward kinematics and the collision check algorithm proposed in (Pan et al., 2012). If the robot motion is collision-free, $u_{k}$ will be added to the search tree and the search process will transit to the next state $x_{k+1}$ at the timestep $t_{k}+{\hat{T}}_{k}$ (see the green block in Figure 3). The planning pipeline repeats until the goal state is reached.

#### 4.4.4 Deploying robot motion to robot system

The robot motions planned by the proposed approach are in a general format of high-level motion commands. Because the robot programming must follow robot manufacture-specific programming rules, the general format of high-level motion commands should be converted to robot manufacturer-specific programming language using a post-processor. Then the robot programs can be directly uploaded to the robot control system. It should be noted that the post-processing here is the syntactical conversation, which is different from the post-processing mentioned in Section 1.1.

## 5 Experiment design and implementation

This section reports the experiment settings and the implementation details of the proposed approach.

### 5.1 Experiment setup

We evaluate the proposed approach on two industrial applications: A handling application with two SCARA robots and a machine tending application with one 6-axis heavy-duty robot.

#### 5.1.1 SCARA robot handling application

**FIGURE 5:**
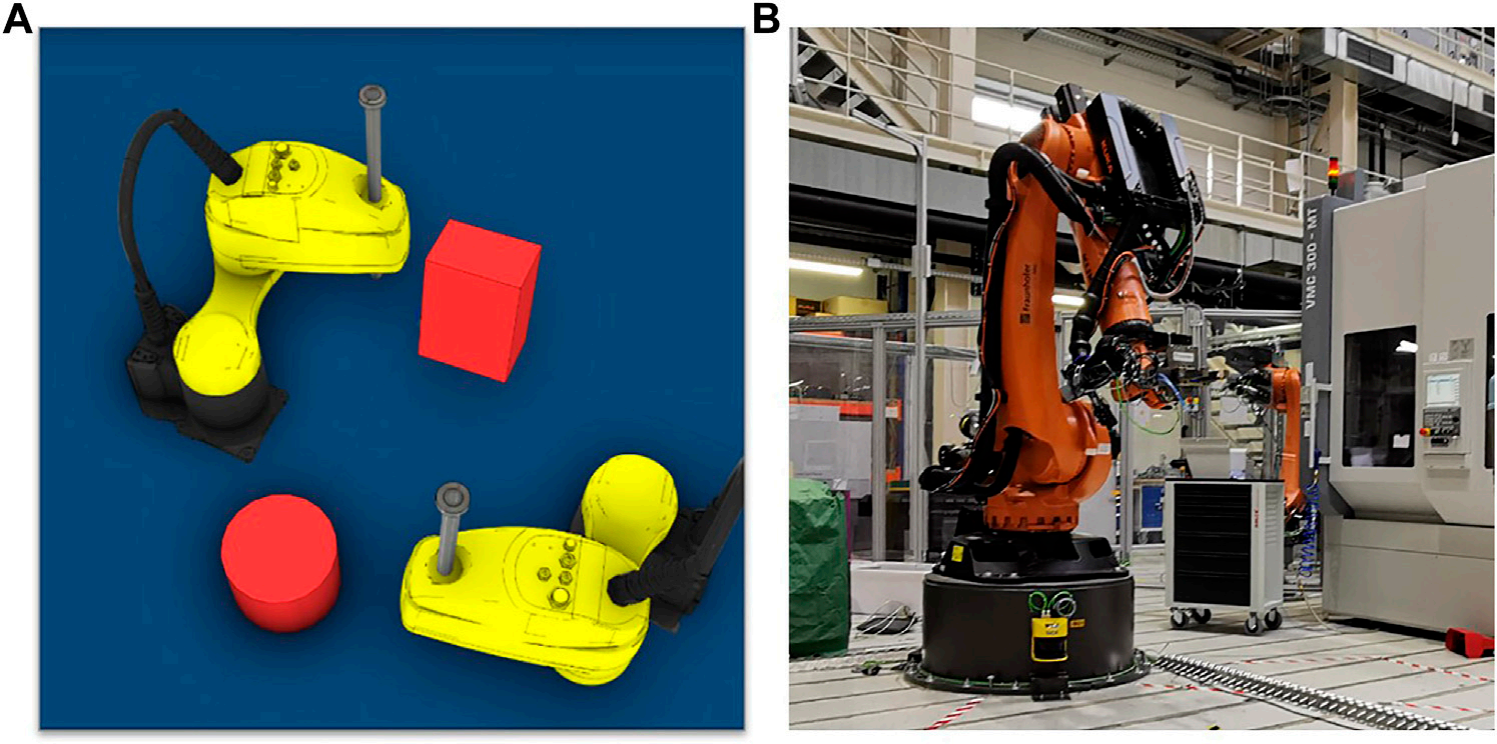
**(A)** SCARA robot handling application (only in simulation environment). **(B)** Machine tending application with a 6-axis heavy-duty industrial robot.

In this application, two SCARA robots perform pick-and-place tasks in different environments containing static and dynamic obstacles. It is important to note that although this application contains two SCARA robots, only the motions of one robot need to be planned, and the other robot is seen as a static or dynamic obstacle. Table 1 details the static and dynamic obstacles in four different categories of environments. In this application, we focus on evaluating the offline planning phase. Thus, the planned motions are only verified in the simulation environment (Figure 5A).

**TABLE 1:** Four categories of environments of the SCARA robot handling application used in experiment.

Categories of environments	Static obstacles	Dynamic obstacles
Simple Staitc Environments	1x Cylinder or Cubic	0
	1x Robot	
Complex Static Environments	3x Cylinders or Cubics	0
	1x Robot	
Simple Dynamic Environments	1x Cylinder or Cubic	1x Roboter
Complex Dynamics Environment	3x Cylinders or Cubics	1x Roboter

#### 5.1.2 Machine tending application

This application evaluates the proposed approach for the problem domain of high-dimensional motion planning. A 6-axis heavy-duty robot loads and unloads a machine tool. Unlike the SCARA robot handling application, the planned robot motion in this application will be deployed and verified on a real robot-based machine tending system to evaluate the complete pipeline from planning until deploying robot motions (Figure 5B).

### 5.2 Implementation

This section describes the structure of neural network models and the datasets.

#### 5.2.1 Dynamic Environment Representation Network

The Dynamic Environment Representation Network uses the basis struct of Variational Autoencoders (VAE) (Kingma and Welling, 2014) with five 3D-CNN layers (Ji et al., 2013) to compress the static obstacles in the environment into a 20-dimensional embedding. For the dynamic environment, for example, in SCARA robot handling application, a 3-layer RNN encoder with ten units to embed the changes of the dynamic environment over time. Each of the ten units accepts a 20-dimensional embedding of one frame of the dynamic environment and the RNN encoder finally produces an embedding vector of the dynamic environment. (See the blue block in Figure 3).

For the SCARA robot handling application, we randomly generate 1000 environments. Each environment contains a varying number of static cylindrical or cubic obstacles and a SCARA robot seen as a dynamic obstacle. Then we recorded a frame of the dynamic environment every 50 milliseconds. The environment in each frame is voxelized and fed into VAE to produce an environment embedding. We take ten frames of environment embedding following the current timestep as a data tuple for training the RNN encoder.

For the machine tending application, 500 static environments are generated. In each environment, we select one of five different machine tools and place it randomly within the reachable workspace of the robot.

#### 5.2.2 High-level control policy network

A high-level motion command consists of the motion type and motion parameters. In this work we consider Point-to-Point (PTP) and Linear motion of the SCARA robot and the 6-axis robot. The motion parameters of both motion commands are the motion speed $v_{k}$ and the state $x_{k+1}$ to be reached.

The High-level Control Policy Network contains two branches: one generates motion type (Motion Type Prediction Branch) and the other generates motion parameters (Motion Parameter Prediction Branch). These two branches take the same inputs: the goal state $x_{Goal}$ , the current state $x_{k}$ and the environment embedding $z_{t_{k}}$ at search step k .

The Motion Type Prediction Branches for the SCARA robot handling and the machine tending applications consist of 10 and 12 fully connected hidden layers followed by a Softmax layer with a two-dimensional output, respectively. The Motion Parameter Prediction Branch is a 12-layer forward neural network for the SCARA robot handling application and a 15-layer forward neural network for the machine tending application, respectively. Unlike the network structure that generates samples at every search step in (Bdiwi et al., 2018), we do not use dropout layers to achieve stochasticity in the Motion Parameter Prediction Branch because the dropout layer affects the convergence of the neural network. Inspired by the struct of VAE, we applied two hidden layers before the output layer to generate two vectors simultaneously: means and standard deviations vector of motion parameters. The output layer samples a final prediction of motion parameters from the means and standard deviations. (See the green block in Figure 3).

In the environments generated in Section 5.2, we collect data for training High-level Control Policy Network. Each environment of the SCARA robot handling application and the machine tending application contains 50 start-goal pairs. In order to make the data set closer to the real machine tending applications, the start position or the goal position of each start-goal pair must be located over the working table inside the machine tool.

**FIGURE 6:**
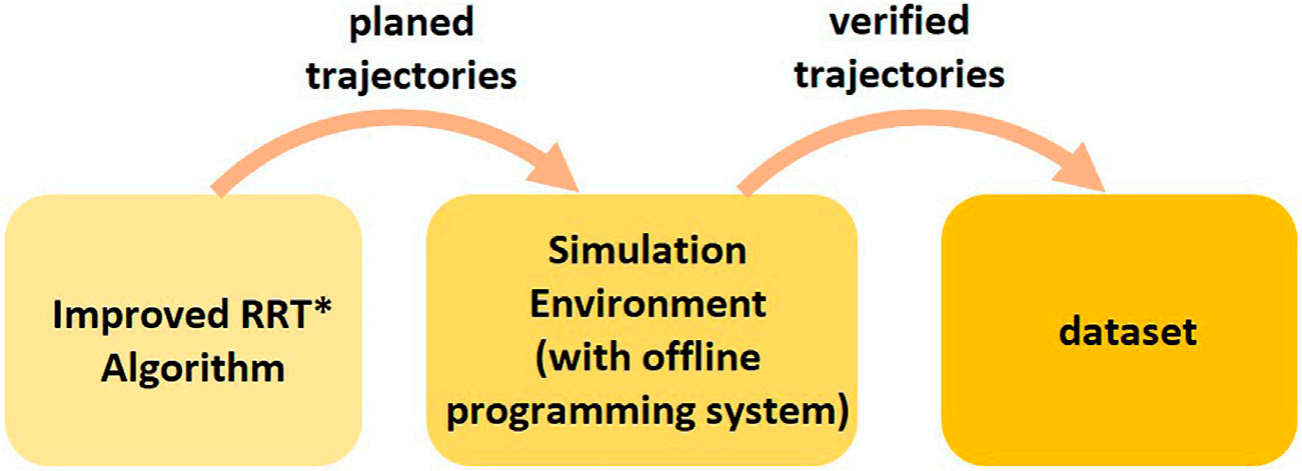
Procedure of collecting dataset for High-Level Control Policy Network.

An improved RRT* approach (Otto et al., 2021) is used to plan a trajectory as expert data. Unlike the basic RRT, the improved RRT* approach post-processes the planned robot motions using PTP and Linear interpolation and generates containing high-level motion commands. The robot motions post-processed by the improved RRT* algorithm may collide with static and dynamic obstacles during the execution due to the inaccurate estimation of robot dynamics and control parameters in the planning phase. Therefore, we execute all generated trajectories in the simulation environment Visual Components with the VRC module and only add the collision-free trajectories and corresponding environment models to the training set (Figure 6).

Both branches of the proposed network are trained in a supervised fashion. The loss of the first branch $L_{T}\left(\theta \right)$ is defined as:

$$\begin{array}{c} L_{T}\left(\theta \right)=-\sum_{i=1}^{2}c_{i}\log\left(p_{i}\right) \end{array}$$ (9)

where i indicates the category of motion command type c and $p_{i}$ represents the predicted probability of the command type $c_{i}$ .

The loss of the second branch $L_{P}\left(\theta \right)$ is defined as:

$$\begin{array}{c} L_{P}\left(\theta \right)=\left|\left|{\hat{v}}_{k}-v_{k}\right|\right|+\left|\left|{\hat{x}}_{k+1}-x_{k+1}\right|\right| \end{array}$$ (10)

where ${\hat{v}}_{k}$ and ${\hat{x}}_{k+1}$ are predicated motion parameters. $v_{k}$ and $x_{k+1}$ are the corresponding ground truth. We use adam optimizer (Kingma and Adam, 2015) with initial learning rate .001, momentum .9. The learning rate is decreased by half every 50 epochs.

#### 5.2.3 High-Level Controlled Robot Dynamics Network

High-Level Controlled Robot Dynamics Network has two branches, the Interpolation State Prediction Branch and Execution Time Prediction Branch, to predict the interpolation states and execution time of realistic robot motion.

The Interpolation State Prediction Branches consist of 12 and 14 fully connected hidden layers for the SCARA robot handling and the machine tending applications, respectively. The Execution Time Prediction Branch consists of 10 and 11 fully connected hidden layers for the SCARA robot handling and machine tending applications, respectively. The Interpolation State Prediction Branch takes the current state $x_{k}$ and motion command $c_{k}$ with motion parameter ( $v_{k}$ and $x_{k+1}$ for Point-to-Point and Linear motion) as input to predicate the execution time ${\hat{T}}_{i}$ . A given interpolation step $t_{k}+n\nabla t$ along with the same input as Interpolation State Prediction Branch is fed into the Execution Time Prediction Branch to predict the interpolation state of the robot ${\hat{x}}_{k,n}$ at the given interpolation step $t_{k}+n\nabla t$ (See the orange block in Figure 3).

The VRC Modul in Visual Components executes ten thousand motion commands of the SCARA robot and fifteen thousand motion commands of the 6-axis heavy duty robot. The execution time and the interpolation states of executed motion commands are recorded as the dataset.

The first and second branches are trained by using standard L2 loss function $L_{Interpolation}\left(\theta \right)$ and $L_{ExecutionTime}\left(\theta \right)$ , respectively:

$$\begin{array}{c} L_{Interpolation}\left(\theta \right)=\left|\left|{\hat{x}}_{k,n}-x_{k,n}\right|\right| \end{array}$$ (11)

$$\begin{array}{c} \;L_{ExecutionTime}\left(\theta \right)=\left|\left|{\hat{T}}_{k}-T_{k}\right|\right| \end{array}$$ (12)

where $x_{k,n}$ and ${\hat{x}}_{k,n}$ denote the ground truth and prediction of robot state at the interpolation step n , respectively. $T_{k}$ and ${\hat{T}}_{k}$ denote the ground truth and prediction of execution time, respectively. During training, we use stochastic gradient descent (SGD) [35] with initial learning rate .0005 and momentum .8.

## 6 Result and discussion

For each application, this section evaluates the proposed approach in 100 new environments, which are not used in the training phase. In each environment, 20 pairs of start and goal were randomly generated. The performance of the RRT, the improved RRT* and the proposed approach was analyzed in terms of validity, the execution time of trajectory and computation time.

### 6.1 Validity of trajectory

#### 6.1.1 SCARA handling application

**FIGURE 7:**
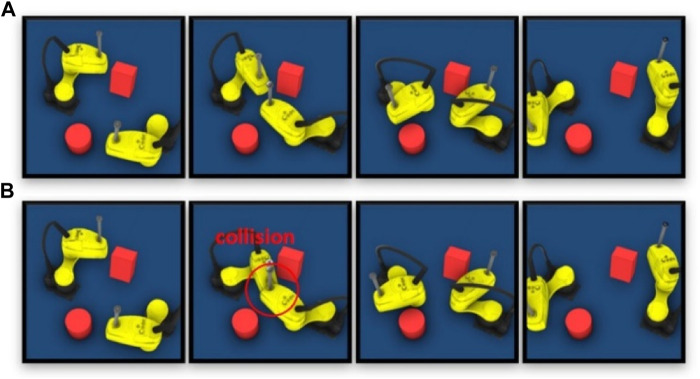
**(A)** Planned trajectory without collision. **(B)** Executed trajectory with collision.

For the SCARA handling application, the robot motions planned offline by different planners are only verified in Visual Components. Figure 7 shows an example of an invalid trajectory generated by RRT. Figure 7A shows that when the SCARA robot follows the planned trajectory exactly, the robot on the right side passes the shared area before the robot on the left side. The executed motion of the robot is slower than computed in the planning phase and enters the shared area later than planned, resulting in a collision with a cubic obstacle (Figure 7B).

In all scenarios of SCARA handling application, only 5.2% of the trajectories generated by our approach are invalid because the trained High-Level Controlled Robot Dynamics Network can predicate the robot motion more accurately in the planning phase. Table 2 shows the relative error of the trained model in predicting motion interpolation and execution time. In all experiment scenarios, the average error between the actual and predicted execution time of the high-level motion commands is 5%. Furthermore, Table 2 shows that the error in predicting Point-to-Point motion is smaller than that in predicting linear motion. The reason is that predicting the dynamics of linear motion requires estimating the inverse kinematic model, which increases the prediction error.

**TABLE 2:** Average prediction error of High-Level Controlled Robot Dynamics Network. The error in predicting execution time is defined as $\frac{{\hat{T}}_{k}-T_{k}}{T_{k}}$ , where ${\hat{T}}_{k}$ and $T_{k}$ are predicted execution time and actual execution time, respectively. The error in predicting motion interpolation is defined as $\frac{\sum_{0}^{i}\left|\left({\hat{x}}_{k,i}-x_{k,i}\right)\right|}{l_{k}}$ , where ${\hat{x}}_{k,i}$ and $x_{k,i}$ are prediction and ground truth of robot state at interpolation step of robot motion, respectively. $l_{k}$ is the euclidean distance along the robot motion executed. Because the 6-axis robot in the machine tending application does not need to avoid other dynamic obstacles in the machine tending application, the robot moves with 100% velocity override to achieve the shortest cycle time.

Motion speed override		Average prediction error of execution time		Average prediction error of interpolation	
		PTP motion (%)	Linear motion	PTP motion (%)	Linear motion
SCARA robot handling application	0 - 25%	2.7	4.6%	.23	.57 %
	25% - 50%	2.9	5.8%	.47	.79 %
	50% - 75%	4.3	6.6%	.48	.86 %
	75% - 100%	6.0	7.2%	.65	.91 %
Machine Tending Application	100%	1.4	—	.31	—

#### 6.1.2 Machine tending application

For the machine tending application, we deployed the robot motions planned offline on the real robot in different ways. The proposed approach generates high-level motion commands that can be directly uploaded to the robot control system (see Figure 2C). Because the improved RRT* uses an interpolation algorithm to convert the planned robot motion to high-level motion commands, the generated motion commands can also be uploaded into the robot control system (see Figure 2B). The RRT generates low-level control inputs, which should be executed in an additional control loop (see Figure 2A).

**FIGURE 8:**
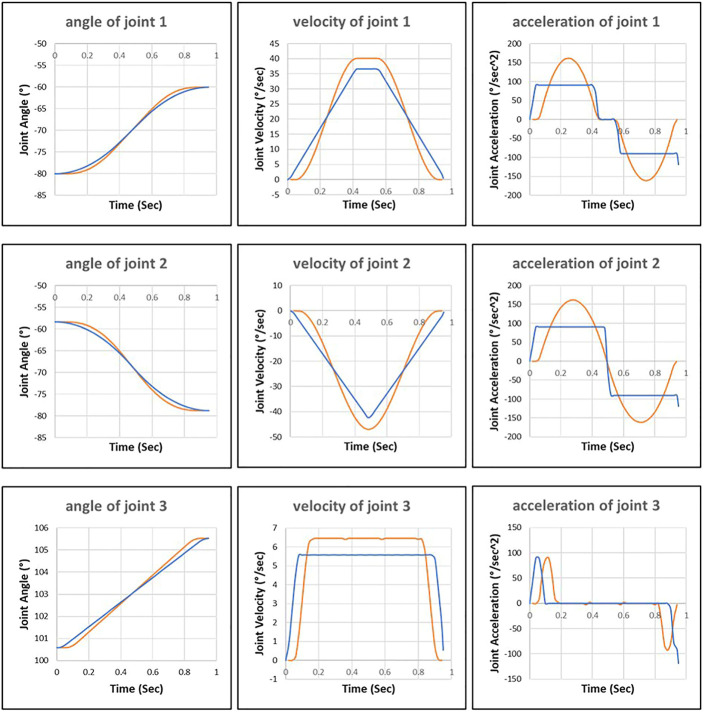
Joint motion planned by the improved RRT* approach (blue line) and joint motion executed by robot control system (orange line).

In Figure 8, we can see that the robot motion planned by the improved RRT* deviates significantly from the robot motion executed by the control system. It is because the control algorithm of the improved RRT* used in the planning phase differs from the control algorithm used in the robot control system-in the offline planning phase, the improved RRT* assumes that the joints can reach the maximum acceleration. However, in reality, the robot control system only applies 60% and 45% of the maximum acceleration to the first and second joints, respectively.

**FIGURE 9:**
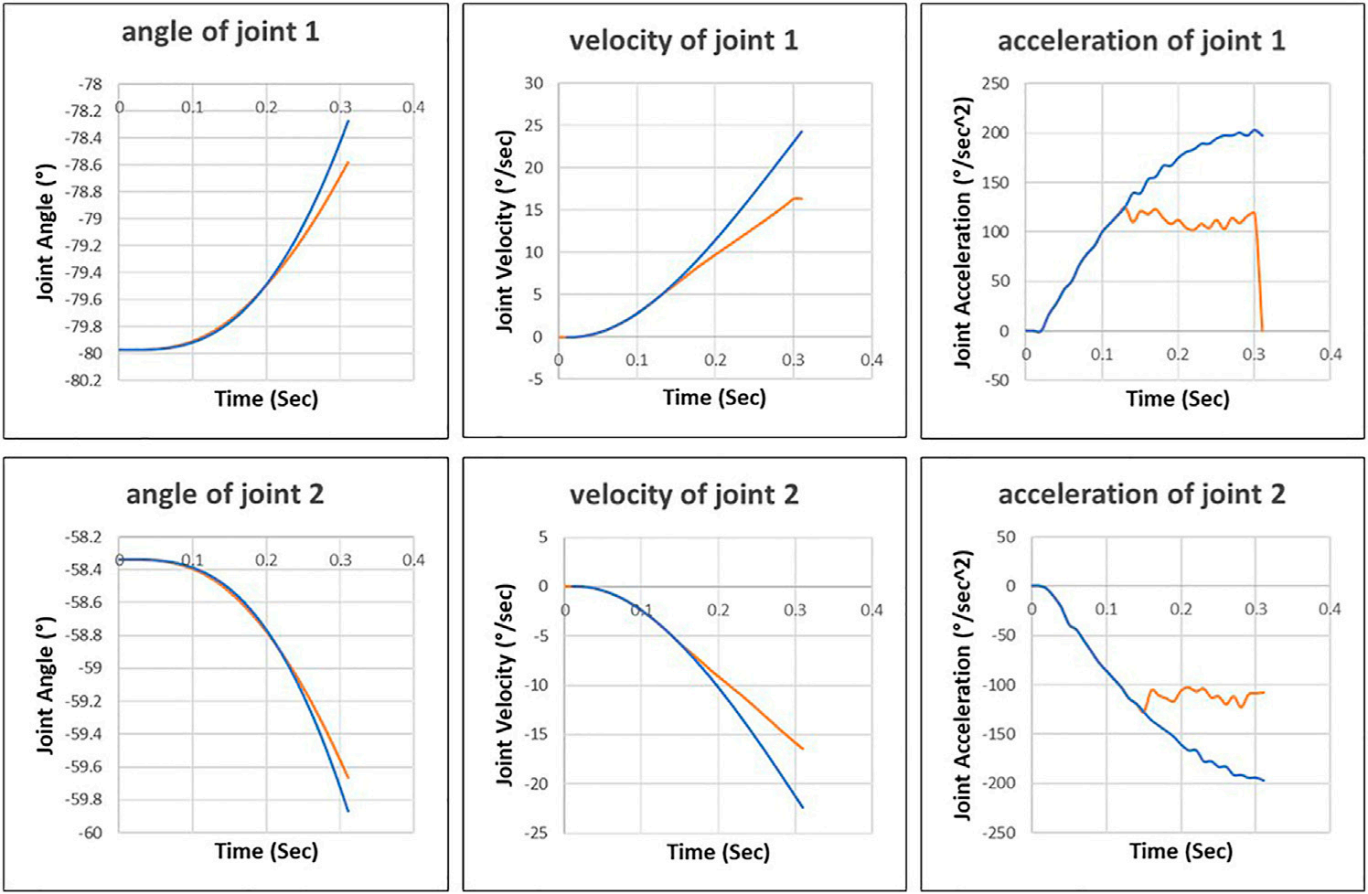
Control inputs of external control loop (blue line) and actual joint motion (orange line).

The control inputs and actual values are recorded during the execution of robot motion controlled by the additional control loop (Figure 9). The additional controller tries to drive the first and second joints with maximum acceleration, but the internal motor controller limits the joints to reach the maximum value. Then the fluctuation of joint acceleration triggers the safety mechanism of the robot control system, which disconnects the communication interface (Robot Sensor Interface) between the additional controller and the robot control system.

**FIGURE 10:**
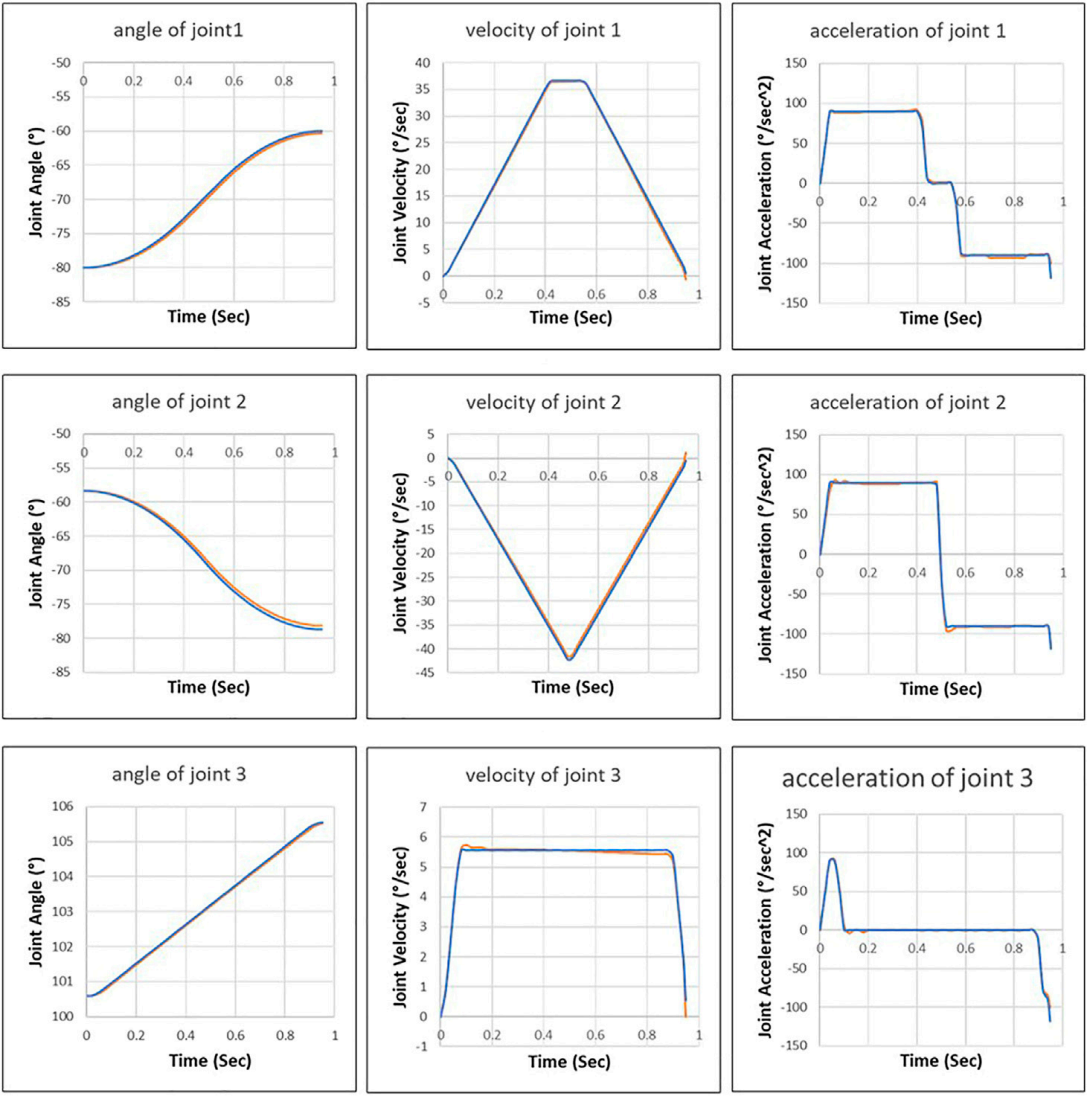
Joint motion planned by the proposed approach (blue line) and joint motion executed by robot control system (orange line).

Figure 10 shows that the robot motion planned by the proposed approach is close to the motion executed by the robot control system. We can see that the trained neural network has learned the control behavior (acceleration and deceleration) of the robot control system to predicate the interpolation of robot motion.

### 6.2 Execution time of trajectory

We compared the execution times of the trajectories generated by the proposed approach, RRT and improved RRT* (Table 3).

**TABLE 3:** Average execution time of trajectories generated by proposed approach and the benchmark approaches.

Environment	Distance between start and goal	Average execution time in second		
		Proposed approach	RRT	Improved RRT*
Simple static environment of SCARA robot handling application	Near	.223	.210	.212
	Middle	.420	.544	.513
	Far	.661	.837	.702
Complex static environment of SCARA robot handling application	Near	.296	.370	.306
	Middle	.605	.801	.664
	Far	.736	.909	.759
Simple dynamic environment of SCARA robot handling application	Near	.246	.276	.266
	Middle	.495	.593	.563
	Far	.733	.948	.829
Complex dynamic environment of SCARA robot handling application	Near	.420	.464	.467
	Middle	.766	.978	.827
	Far	1.070	1.292	1.116
Machine tending application	Near	1.523	1.892	1.328
	Middle	2.034	2.367	2.249
	Far	3. 551	3.719	3. 406

It is necessary to note that the trajectory’s execution time varies significantly due to different distances between the start and goal states. To compare the performance of different approaches more reasonably, we classify the planning tasks into three categories according to the distance between the start and goal states: 1. near distance (smaller than 30% of the robot’s range), 2. middle distance (bigger as 30% but smaller as 60% of the robot’s range) and 3. far distance (bigger as 60% of the robot’s range). It can be seen that the average execution time of the trajectories generated by the proposed approach is twenty percent faster than RRT in the SCARA robot handling application. Since improved RRT* optimizes the number of *via* points while expanding the search tree, the execution time of the trajectories generated by it is essentially the same as the proposed approach. However, the optimization increases computation time, as seen in section 6.3. Since all scenarios of the machine tending application are simple, the execution time of the motion planned by each approach varies slightly.

**FIGURE 11:**
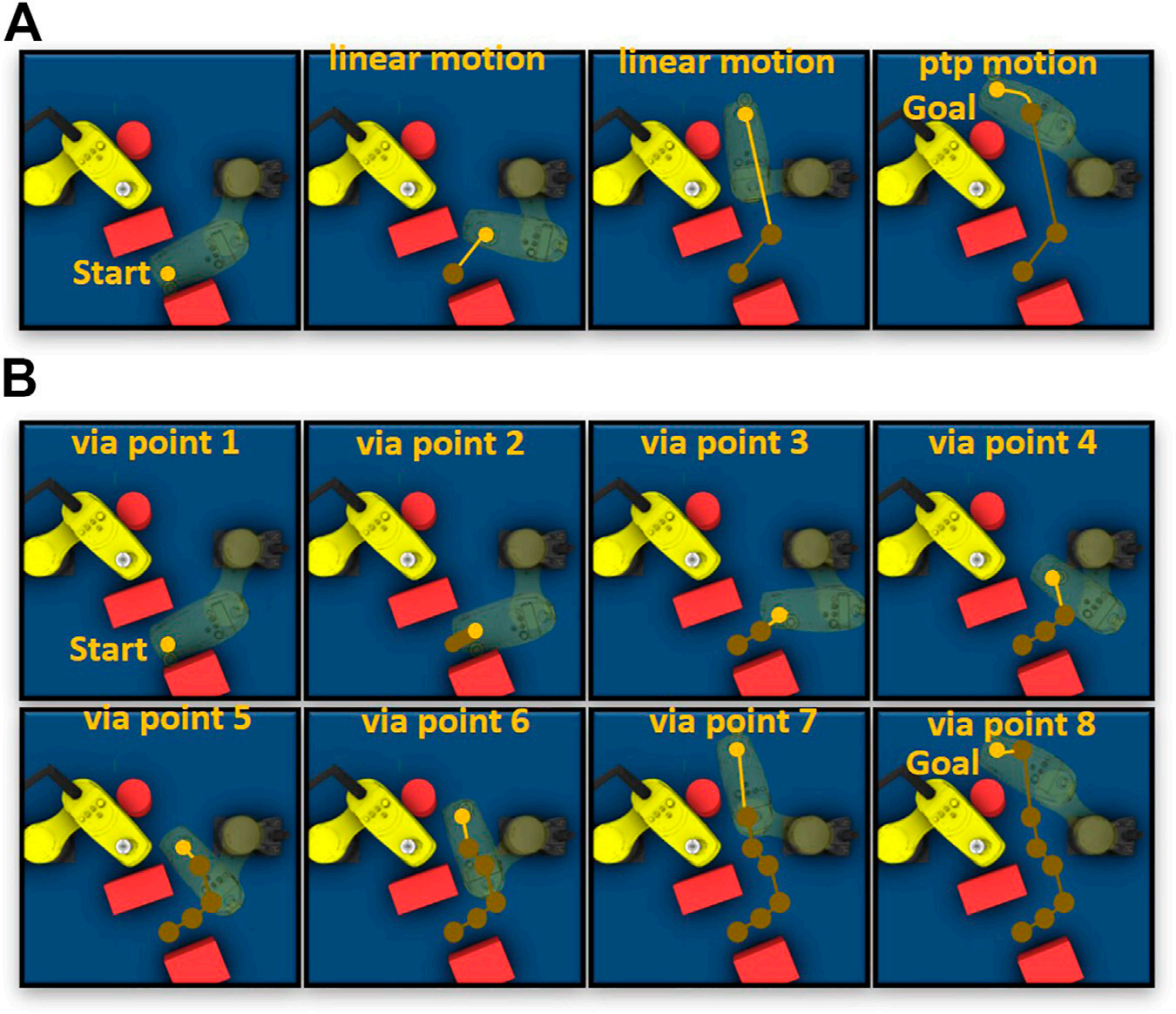
**(A)** Example trajectory generated by the proposed approach. **(B)** Example trajectory generated by RRT. The green points represent the *via* points of trajectory in Cartesian space.

Figure 11 shows the valid trajectories generated by the proposed approach and RRT in an example scenario. The proposed approach generates a trajectory containing only three high-level motion commands (Figure 11A). The first and second linear motion commands guide the robot through a narrow area. After the robot leaves the narrow area, the proposed High-level Control Policy Network maps the empty surrounding area to a Point-to-Point motion command because the Point-to-Point motion is faster than the linear motion. RRT generates more *via* points (Figure 11B) in the narrow area, resulting in acceleration and deceleration of robot joints.

### 6.3 Computation time

**FIGURE 12:**
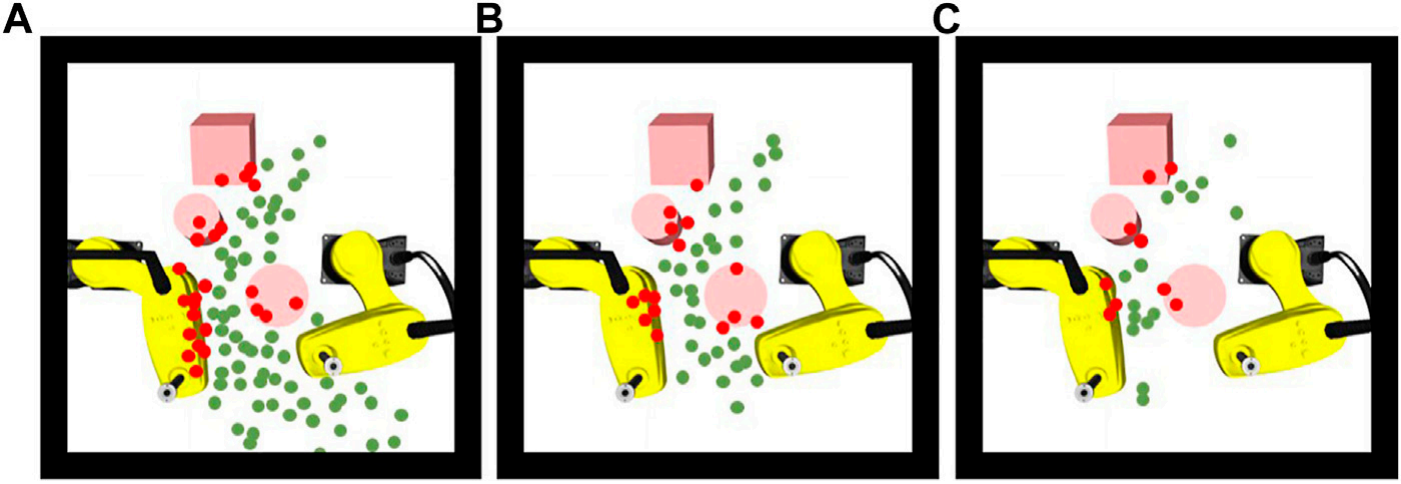
**(A)** Valid (green) and invalid (red) samplers generated by RRT, **(B)** RRT* and **(C)** by the proposed approach.

We compared the computation time of the proposed approach with the benchmark approach in scenarios with different complexities. As the environment becomes more complex, the advantage of our approach in terms of computation time becomes obvious (Table 4). In particular, the proposed approach is up to 10 times faster than the improved RRT* approach in complex dynamic environments of SCARA robot handling application because the proposed approach reduces the computation time by efficiently exploring in learned feasible solution space. In Figure 12, we visualize all the samples generated by the different approaches for the same task. It has been found that the benchmark approaches spent much time to generate a large number of samples randomly. The proposed approach generates fewer samples in critical areas based on environment information.

**TABLE 4:** Average computation time of trajectories generated by the proposed approach and benchmark approaches.

Environment	Distance between start and goal	Average computation time in second		
		Proposed approach	RRT	Improved RRT*
Simple static environment of SCARA robot handling application	Near	.18	.37	1.82
	Middle	.26	.61	2.27
	Far	.31	.84	2.38
Complex static environment of SCARA robot handling application	Near	.62	1.50	2.47
	Middle	.74	2.81	4.77
	Far	.88	4.16	5.49
Simple dynamic environment of SCARA robot handling application	Near	.42	1.83	5.05
	Middle	.57	3.59	6.92
	Far	.59	4.24	7.22
Complex dynamic environment of SCARA robot handling application	Near	.67	3.12	5.34
	Middle	.89	5.94	8.03
	Far	.94	7.12	9.11
Machine tending application	Near	.90	.85	1.15
	Middle	1.08	.88	1.27
	Far	1.17	.92	1.63

## 7 Conclusion

We have proposed a novel deep neural network that generates collision-free trajectories as high-level motion commands. The generated trajectory can be directly deployed in the robot control system without post-processing. Furthermore, the experiment results show that the proposed approach outperforms the benchmark approaches in terms of validity, execution time of planned motion and computation time. One future direction is to extend our data collection procedure and generalize our network to handle more high-level commands for robots with higher degrees of freedom.

## Data Availability

The datasets presented in this article are not readily available because NDA is necessary. Requests to access the datasets should be directed to shuxiao.hou@iwu.fraunhofer.de.
